# Regenerative Strategies for Retinal Neurons: Novel Insights in Non-Mammalian Model Organisms

**DOI:** 10.3390/ijms23158180

**Published:** 2022-07-25

**Authors:** Elisabetta Catalani, Agnese Cherubini, Simona Del Quondam, Davide Cervia

**Affiliations:** Department for Innovation in Biological, Agro-Food and Forest Systems (DIBAF), Università degli Studi della Tuscia, 01100 Viterbo, Italy; agnesecherubini@live.it (A.C.); simonadelquondam@outlook.it (S.D.Q.)

**Keywords:** neurodegeneration, neuronal regeneration, retina, zebrafish, *Drosophila melanogaster*

## Abstract

A detailed knowledge of the status of the retina in neurodegenerative conditions is a crucial point for the development of therapeutics in retinal pathologies and to translate eye research to CNS disease. In this context, manipulating signaling pathways that lead to neuronal regeneration offers an excellent opportunity to substitute damaged cells and, thus, restore the tissue functionality. Alternative systems and methods are increasingly being considered to replace/reduce in vivo approaches in the study of retina pathophysiology. Herein, we present recent data obtained from the zebrafish (*Danio rerio*) and the fruit fly *Drosophila melanogaster* that bring promising advantages into studying and modeling, at a preclinical level, neurodegeneration and regenerative approaches in retinal diseases. Indeed, the regenerative ability of vertebrate model zebrafish is particularly appealing. In addition, the fruit fly is ideal for regenerative studies due to its high degree of conservation with vertebrates and the broad spectrum of genetic variants achievable. Furthermore, a large part of the drosophila brain is dedicated to sight, thus offering the possibility of studying common mechanisms of the visual system and the brain at once. The knowledge acquired from these alternative models may help to investigate specific well-conserved factors of interest in human neuroregeneration after injuries or during pathologies.

## 1. Introduction

Cell death, inflammation and oxidative stress are the foremost common mechanisms occurring in degenerative diseases of the central nervous system (CNS), including those regarding the retina and the visual system [1,2]. New strategies and approaches targeting these general pathological aspects are continuously evaluated, and particular attention is paid to early biomarkers [2]. Retinal neurodegenerative diseases are the principal cause of vision impairment and vision loss, affecting people globally. The retina, anatomically and developmentally, is known as an extension of the CNS and displays similarities to the brain and spinal cord also in terms of response to insult, immunology, and neurodegenerative manifestations [3,4]. For example, glaucoma, a group of diseases characterized by progressive optic nerve degeneration and irreversible blindness, can be considered a neurodegenerative disorder of both the eye and the brain [5]. Indeed, glaucoma shares common neurodegeneration features with amyotrophic lateral sclerosis, Parkinson’s disease, Alzheimer’s disease, and other tauopathies, such as chronic traumatic encephalopathy and frontotemporal dementia. Intriguingly, the structural and functional damage of retina may be useful for the early diagnosis of CNS diseases, as retinal defects often precede pathological signs in the brain [3,4]. Modern imaging techniques can be used as a non-invasive method to check the retinal status, thus offering the possibility of observing vascular, structural, and functional aspects in humans commonly related to a particular brain disease [3,6].

Establishing links between the retina impairments and neurodegenerative diseases appears particularly challenging for clinical strategy’s future development, and there is an urgent need for innovative technologies and standardized methodologies. It is noteworthy that a detailed knowledge of the status of the retina in neurodegenerative conditions is a crucial point to translate eye research to CNS disease. It is well established that neurodegeneration of the visual system reflects a complex scenario as several components contribute, i.e., neurons, vessels, inflammatory cells, immunological features, and biomechanical impairments [2]. Environmental factors, metabolic stress, neurovascular coupling, and genetic backgrounds may also play a fundamental role in retinal neurogenerative disorders, thus representing additional target areas to study for therapeutic interventions [2]. Many possible treatments are under evaluation for retina neurodegeneration, including gene therapy, antioxidants, anti-inflammatory, and antiapoptotic substances, alone or in combination [1]. An intriguing perspective is the possibility of regeneration after injuries [2]. For instance, there are exciting suggestions to identify factors that could be crucial for driving the regeneration of the RGC axon. The manipulation of systems such as stem cell-derived oligodendrocytes, intrinsic RGC-specific factors (the lipid phosphatase PTEN and the suppressor of cytokine signaling 3), transcription factors, extrinsic factors (i.e., mammalian target of rapamycin (mTOR)-activating proteins), and growth factors is a promising strategy for the survival and regenerative potential of RGC [2]. In addition, the inflammatory response is a condition that can promote the regeneration of axons [7], thus representing an exceptional opportunity to improve the management of retinal neurodegenerative pathologies.

The development of several therapeutics in retinal diseases has been facilitated by decades of research into the cellular and molecular mechanisms using either human cell-derived 2D systems and animal models. However, advances in alternative systems and methods are increasingly being considered worldwide to replace, or at least reduce, in vivo approaches (especially on mammals) in biomedical fields, including the study of retina physiopathology. Here, we reviewed recent data obtained from the zebrafish (*Danio rerio*) and the fruit fly *Drosophila melanogaster* that bring promising advantages into studying and modeling, at the in vivo preclinical level, neurodegeneration and regenerative approaches in retinal diseases.

## 2. Alternative Organism Models for Retina Neuroregeneration

Unlike in vitro cell cultures that cannot mimic tissue homeostasis and physiology, 3D retinal organoids are relatively cheap models and have an undeniable complexity rate [2,8,9]. However, they are challenging to isolate and maintain long enough to investigate complex processes such as inflammation and neovascularization. These disadvantages are exacerbated considering the retina, which is mainly due to the global complexity of this tissue. For instance, the organotypic cultures lack blood flow and biomechanical support. Of interest, organ culture avoids using a high number of animals and permits a straightforward therapeutic approach. Indeed, several classic diagnostic techniques could be applied to retinal organ cultures, such as optical coherence tomography, which explores the morphological aspect of the retinal architecture, electroretinograms that record the electrical response of retinal cells, and microelectrode array recording, which stimulates and records the electrical activity of RGC. Several mammalian retinal organ cultures as alternative models are currently available and well established, including those derived from mice, rats, rabbits, cats, dogs, non-human primates, bovines, and pigs [9,10]. They are excellent samples for the preliminary phase before the in vivo step and for therapy tests, although organ cultures for the study of complex retinal neurodegenerative pathologies such as diabetic retinopathy (DR), retinitis pigmentosa (RP), age-related macular degeneration, and glaucoma are not entirely reproducing the human condition [2,8,9]. Although all the events occurring during the various steps of retinal neurodegenerative diseases, including the clinical progression, are not fully mimicked by a single animal, preclinical in vivo models provide important information on the molecular and cellular mechanisms at the basis of the neuronal impairment. Thus, multiple organisms, including non-mammalian ones, are crucial for validating the mechanisms involved in retinal pathologies and developing new therapeutic options.

### 2.1. Zebrafish to Gain Insight in Vertebrate Retina

**Figure 1:**
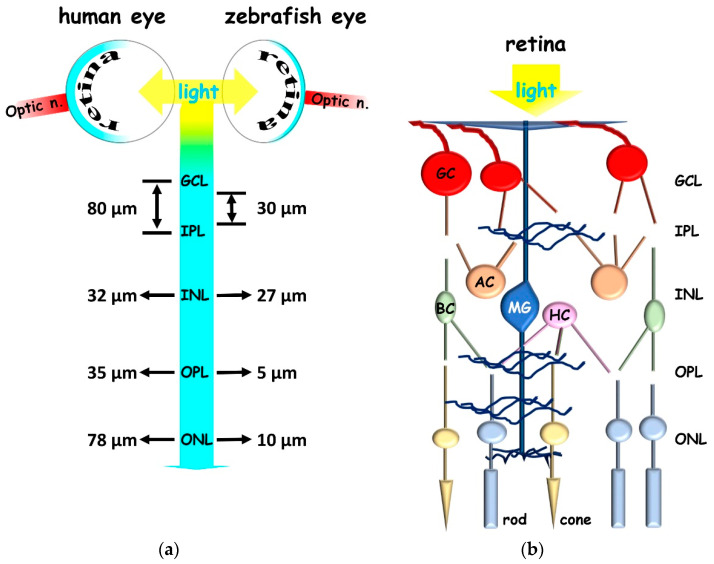
Schematic representation of human and zebrafish retina. (**a**) Retinal layers thickness. (**b**) Retinal layers and the main neuronal types. GCL (ganglion cellular layer); IPL (inner plexiform layer); INL (inner nuclear layer); OPL (outer plexiform layer); ONL (outer nuclear layer); GC (ganglion cell; red); AC (amacrine cell; orange); BC (bipolar cell; green); MG (Müller glia; blue); HC (horizontal cell; pink); rod (light blue), cone (yellow), optic n. (optic nerve).

Zebrafish retina can regenerate after injury and is considered an ideal model for dissecting mechanisms relevant to retinal disease management [11]. Compared to humans, the zebrafish vision system shares structural and functional similarities (Figure 1) [12]; for example, it is cone-dominated, since zebrafish is a diurnal animal. Like in humans, the zebrafish retina consists of three nuclear layers (outer, inner, and RGC layer) containing neuronal soma separated by two plexiform layers (inner and outer) where synapsis takes place [13]. Photoreceptors consist of one rod cell type and blue and red–green cone types. In addition, zebrafish retina contains UV-sensitive cones, which are missing in humans. Zebrafish is a vertebrate with good color vision and high visual acuity [14]. RGC bodies are located in the RGC layer, while inner neurons consist of amacrine, horizontal, and Müller glial (MG) cell bodies, which are a type of retinal stem cell responsible for regenerative responses [15]. MG are radial glial cells in the inner vertebrate retina, which have a cylindrical, fiber-like shape. After an injury, MG de-differentiate and start asymmetric divisions that lead to the production of cells with glial properties and neuronal progenitor cells that proliferate, migrate and differentiate into new neuronal cell types [15]. Zebrafish possess tremendous intrinsic regenerative potential in ocular tissues, including the retinal pigment epithelium (RPE), while mammalian RPE is limited in its regenerative capacity. RPE inflammatory events highly participate in neurodegenerative progress, and RPE dysfunction or disease can lead to blindness in humans [16]. Of interest, macrophage/microglia cells have been recently shown to be responsive to RPE damage in zebrafish, and their function is required for the timely progression of the RPE regenerative response [17]. Several experimental methods could be used to induce injury in the zebrafish retina, and numerous factors/pathways are responsible for the activation of neuroprotective/regenerative ability, including neurotrophic and growth factors, the Janus kinase/signal transducer and activator of transcription (JAK/STAT, which directly controls reprogramming genes), Wnt (which is implied in stem fate decision), sonic hedgehog (Shh, which is crucial for cellular differentiation), and the glutamate receptor NMDA signaling [11]. A set of experiments has been recently performed using the adult zebrafish retina, demonstrating that a light lesion results in a loss of photoreceptors and severe vision impairment that is fully restored within 28 days, but with a gradual recovery [18]. Indeed, the optokinetic response (OKR) behavioral test, a robust test that depends on vision, indicated that functional vision is restored rapidly and gradually upon injury between 7 and 14 days after lesion. In particular, more simple stimuli could be resolved adequately after 10 days, including the vision for high contrast, low level of details, and color vision, while more difficult ones required about 14 days. Furthermore, at 28 days after lesion, OKR tests indicate the complete restoration of vision compared to healthy animals, while morphological recovery is not fully completed, as indicated by photoreceptor mosaic (dys)organization. However, a close correspondence was generally observed between functional and morphological recovery. Thus, although the restoration of visual performance can be observed already before full morphological recovery of the zebrafish tissue, as expected, both structure and function are crucial steps of the regeneration process because newly regenerated cells must fully integrate into the retinal circuitry. The 28 days’ time-course transcriptomic map of zebrafish retinal degeneration/regeneration was recently described [19]. This study highlights that the new progenitor cells derived from MG differentiated into new photoreceptors between 5 and 10 days after vision injury. Most importantly, the trend of transcriptional recovery of opsins and rhodopsin genes revealed details relevant for the comprehension of regenerative retinal process and future applications. As for instance, it suggests that each cone type, and probably opsins within the same cone (red and green opsins are housed within the same double-cone in zebrafish [20]), may retain a kinetically distinct differentiation [19]. To note, 28 days after the lesion, the global transcriptional state did not return to the uninjured condition.

Among the numerous signaling pathways involved in the regenerative potential of the zebrafish retinal neurons, the upregulation of transcription factor achaete-scute complex-like homolog 1 (ASCL1) appeared crucial in MG activation and reprogramming [21]. Indeed, it regulates several regeneration-associated signaling pathways strictly involved in key reprogramming/regenerative retinal steps, including the transcriptional regulator Notch, the transforming growth factor beta (TGF-β), and Wnt/B-catenin. It is noteworthy that Notch and (TGF-β) signaling are negative regulators of reprogramming/regeneration of the MG. Notch maintains MG in a quiescent state around the damaged area to prevent excessive proliferation and block differentiation into new neurons. On the contrary, Wnt/B-catenin signaling is crucial for retinal regenerative processes since it promotes the expression of ASCL1 and the subsequent regeneration response cascade. Furthermore, several other factors activate signaling cascades that lead to the MG reprogramming and regeneration of the injured zebrafish retina. Among them, the hedgehog family (the major regulator for cell differentiation and cell proliferation, including Shh which is critical for retinal regeneration following injury), STAT3 (the signal transducer and activator of transcription 3), α1-tubulin (a neuron-specific microtubule protein), FOXN4 (the forkhead box N4), ZIC2 (the zic family member 2), the transcriptional repressor insm1a, Apobec2b (the apolipoprotein B MRNA Editing Enzyme Catalytic Subunit 2), MAPK (the mitogen-activated protein kinase), PI3K/AKT (the phosphatidylinositol 3-kinase/protein kinase B), PAX6 (a member of the paired box gene family), SOX2 (the sex determining region Y-box 2), MYC (the myelocytomatosis oncogene), OCT4 (octamer-binding transcription factor 4), and RNA-binding protein LIN28, this latter being crucial in inducing pluripotent stem cells [21]. In addition, changes in epigenetics such as DNA methylation, histone modification, and miRNA-mediated degradation of mRNA concur with MG functionality [22], as well as immune response and microglia contribute to retinal regenerative progress mediated by MG [23]. Indeed, microglia cells respond quickly to an injury, which induces an inflammatory reaction promoting MG reprogramming, likely through mTOR signaling.

The stimulation of MG to regenerate injured neurons provides an excellent opportunity to repair degeneration of the retina, which is also associated with aging. In this respect, wild-type zebrafish can live up to 3.5 years in laboratory conditions, and they can accumulate the classic hallmarks of human retinal aging, such as DNA damage, shorter telomeres, and vision decline [24]. Furthermore, retinas of old zebrafish undergo tissue thinning, photoreceptor disorganization and neuronal loss, including RGC and bipolar cells. These morphological alterations occur independently, at least in part, from telomerase, since both wild-type and prematurely aged mutant tert−/− displayed the same scenario. Interestingly, a reduced expression of the crucial molecules is related to the regenerative process and coupled to the altered morphology of MG in aged retinas. In addition, when acute damages occur, aged retinas retained their ability to proliferate into new neurons [24]. These observations suggest that a certain level of key signals, reduced by aging, is necessary for the regenerative processes and manipulating these targets may improve neuroregeneration after injuries as well as in old age, when the already low ability to repair neurons is even more reduced. The main limitations of neural stem cell transplant usage in CNS are the risk of tumors caused by gene mutations and the change of the surrounding environment [25,26]. In addition, reprogramming factors might in some way interfere with normal circuit-based neural functions and might have different effects on different neuronal subtypes [25]. On the contrary, the glial cell-reprogramming approach can have much higher safety and efficiency in generating new neurons than neural stem cells [26]. Stem cell transplants in the human retina are not completely safe yet, and it is difficult to obtain functional and well-integrated neurons [27,28]. It should be noted that unlike zebrafish, mammalian MG regenerative potential is minimal, since they undergo reactive gliosis events, not regenerative processes, after injuries [29]. MG can change their morphology and gene expression but fail the pathway that leads to reprogramming into the major retinal neuron types, and this may be related to distinct neuroregeneration pathways across species. Still, mammalian MG retain the potential to differentiate into retinal neurons under the proper condition, and the future goal will be to understand molecular mechanisms in humans that orchestrate and stimulate these events [29]. Overall, the amount of genetic information available from zebrafish mutants, including evolutionary well-conserved factors associated with the possibility of inducing visual impairment, supports zebrafish as a powerful model for understanding visual disorders. However, besides the exceptional ability of the zebrafish retina to regenerate after injury, MG failed to proliferate and regenerate damaged cells in several zebrafish models: for instance, mutants bbs2 (BBS disorders), MZcep290fb208 (retinal dystrophy), cerkl (nonsyndromic RP and cone-rod dystrophy), eys (autosomal recessive RP), rp2 (X-linked RP) rpgrip1 (RP GTPase regulator interacting protein 1, X-linked RP) [30]. In rats and mice, extensive effort is paid to manipulate MG to induce their regenerative ability and/or reprogramming potential, especially for substitute photoreceptors and RGCs, which are the most affected populations of the mammalian retina during degenerative diseases that cause blindness. However, only a few levels of regeneration based on MG activity were observed after injury, which does not help restore vision broadly [31]. Beyond all these fascinating insights, the in vivo application is still challenging. The complexity of mammalian systems also consists of systemic events, such as the immune response, directly influencing the ability of MG to regenerate lost neurons. Microglia represent the resident innate immune cells of the CNS, which appear dysfunctional during aging due to a constant low-level inflammatory state that is partly responsible for neurodegeneration [32]. Microglia may thus over-react to injury, promoting a prolonged inflammatory response and also causing secondary damages, which does not favor the limited regenerative ability of the CNS. An important aspect to consider during zebrafish regeneration is microglia’s functional and active role, which does not increase during aging in zebrafish, likely reducing the regenerative ability of the aged-retina [24,33].

### 2.2. The Opportunity of D. melanogaster for Neuroregenerative Strategies

**Figure 2:**
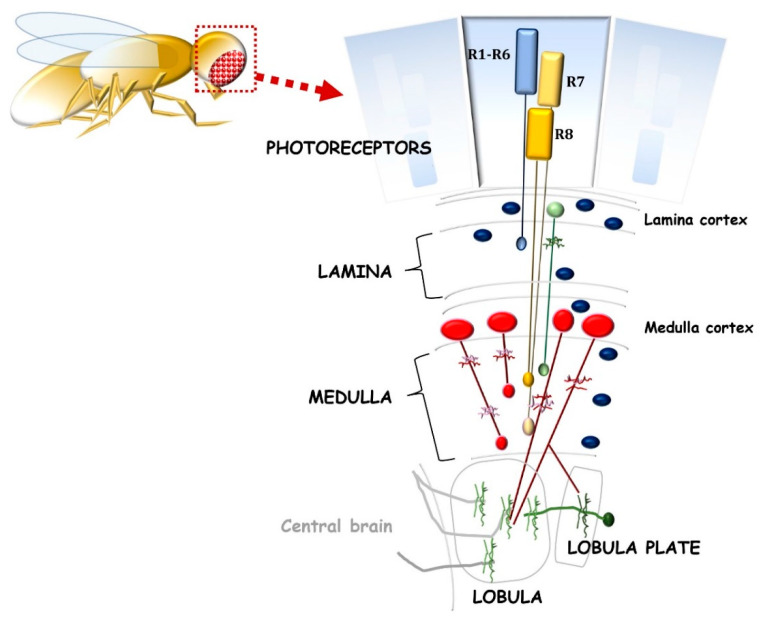
Schematic representation of the drosophila visual system, including the retina and optic neuropils: lamina, medulla, lobula, and lobula plate. Photoreceptors R1–R6 (blue bars) innervate the lamina, and R7–R8 (yellow bars) innervate the medulla. Green, red and blue cells represent lamina neuron, various medullary neurons, and glial cell types, respectively.

Although the drosophila visual system is morphologically and structurally different from the vertebrate one, many parallels can be described (Figure 2). Drosophila captures visual information by the retina and processes it through the optic lobes [34,35]. Each optic lobe consists of four distinct neuropiles: Lamina, Medulla, Lobula, and Lobula plate. The fly retina contains photoreceptors (R1–R8) that project their axons into the optic lobes. In particular, R1–R6 synapses with interneurons in the lamina, while R7 and R8 project to the medulla. These events resemble vertebrate photoreceptors’ synapsis with bipolar cells. In drosophila lamina and medulla, several cell types integrate signals as horizontal and amacrine cells in vertebrates. Furthermore, Lobula cells, such as RGC, send their axons to high-order neurons in the brain. These features suggest that the relevant mechanisms involved in the homeostasis of the retina neurons are well conserved. Remarkably, optic lobes, which project to the central brain, represent more than 60% of the brain. Therefore, in drosophila, a large part of the whole brain is dedicated to sight.

The human CNS cannot establish de novo neurogenesis after injury, but it can probably be induced by manipulating a specific molecular target. Since many basic biological, physiological, and neurological properties are extraordinarily conserved between mammals and *D. melanogaster*, flies represent a well-established system to understand and manage neurogenesis in the mammalian CNS [36], also representing a robust experimental in vivo tool for studying retinal dysfunctions [37]. In this respect, we have recently highlighted the morphological and cellular damage of drosophila retina after hyperglycemia induced by high-sucrose diets, thus offering a meaningful opportunity of using a simple in vivo model to study the pathophysiology of neuroretinal alterations that develop in patients at the early stages of DR [38]. The same fly model has been utilized to counteract eye neurodegeneration and oxidative stress by means of neuroprotective nutraceutical strategies [39]. Similar to mouse models, early retinal neurodegeneration was detected also in two dystrophic mutants of drosophila, i.e., lacking functional large isoforms of dystrophin-like protein, demonstrating that in complex pathologies, such as Duchenne muscular disease, defects of full-length dystrophin trigger retinal neuron damage and synapse alterations [40]. Of interest, we observed the morphological remodeling of the retina in parallel with reduced functionality long before the muscular system appeared compromised.

*D. melanogaster* possesses glial cells in the visual system, which are also present in the entire nervous system, [41]. Like in mammals, glia plays a complex homeostatic role in the nervous system of flies, being in close morphological and functional connection with neurons. During the visual system development of drosophila, glial cells are crucial in mediating neural circuit assembly and forming boundaries [35,42], while in the mature visual system, they have a pivotal role in synaptic transmission and visual processing [41]. In flies, non-neuronal ommatidial cone cells (CC) support retinal neuronal cells and share structural, molecular, and functional aspects with vertebrate MG [43]. CC express specific conserved effector genes of the glial cells, including the pump Na/K-ATPase, the K-inward rectifying channels (Kir channels), the excitatory amino acid transporters (EAAT1), the glucose transporter 1, and the lactate dehydrogenase (LDH). Moreover, CC express prospero and PAX2 transcription factors related to glia functions. In particular, Pax2 is crucial in maintaining a correct retinal structure, while prospero is crucial in supporting photoreceptors, for instance, preventing light-dependent degeneration of photoreceptor cells. It was suggested that CC and inter-ommatidial pigment cells of drosophila eye act as MG and RPE of the vertebrate retina, respectively [43]. This evidence supports *D. melanogaster* as an alternative model that offers the opportunity to manipulate glial cells of the retina to unveil some aspects of MG functions.

Critical aspects of regeneration have been studied in the imaginal disc of flies, which has high regeneration ability after injuries [36]. For instance, oxidative stress occurrence in an injured imaginal disc has been observed under several different signaling, including Nox/Duox NADPH- oxidases [44]. In addition, reactive oxygen species activate and regulate c-Jun N-terminal kinases (JNK), p38 stress-activated MAPK, and the JAK/STAT signaling pathway that could drive the proliferative rate of the environment surrounding the wound, also stimulating drosophila insulin-like peptide (dilp8). Dilp8 is crucial in balancing the developmental delay and the growth of healthy and damaged tissue. JNK signaling was shown to target wg (Wnt1 homolog), which is involved in regeneration, dpp (bone morphogenetic protein decapentaplegic), taking part in growth during imaginal disc development, and hippo signaling [44]. Wg, dpp and hippo are well conserved crucial components in growth and development. Furthermore, it has been shown that JNK downregulates the polycomb group genes during the regenerative process of drosophila imaginal disc. The polycomb group proteins are crucial in maintaining the correct genetic program during reprogramming and cell fate. Of interest, the regeneration of injured imaginal discs has been also positively associated with necrosis-induced apoptosis events [45]. Although the mechanisms involved are not entirely clarified yet, there is evidence that cells involved in necrosis-induced apoptosis did not express mitogen factors detected during the apoptosis-induced regeneration, such as wg and dpp, nor upregulated JNK signaling. In addition, it was observed that signaling pathways activated far from the injured area could be crucial in the regenerative process, such as the metabolism of kynurenine in the fat tissue, which plays a key role in imaginal disc repair [46].

In the central brain of fly larvae, it has been recently demonstrated that P13K and epidermal growth factor receptor (EGFR) signaling activation, mediated by the c-Myc transcription factor, are essential for glia reprogramming and the regeneration of axons after injury [47,48]. Indeed, P13K and EGFR signaling activation induced the upregulation of glycolytic metabolites, such as LDH and L-2GH, which, in turn, promoted axon regeneration. Glycolytic metabolites inactivated γ-Aminobutyric acid receptors type b and incremented cAMP levels, both having a key role in neuronal growth induced by glial reprogramming. Remarkably, regeneration by glycolytic metabolites signaling is retained in mammals. In addition, Notch and the chondroitin sulfate proteoglycan NG2 homolog called Kon promoted glial proliferation in drosophila, similar to vertebrates [49]. In particular, Kon induced glia differentiation and the expression of prospero, thus maintaining proliferation. Prospero was also regulated by negative feedback by neuronal Islet antigen-2 (Ia-2). After an injury, PI3K, Kon, and Ia-2 levels increased, also promoting dlp6 secretion from neurons, which induced glial reprogramming at the end of a complex signaling pathway. Ia-2 and insulin signaling may rewire glial cells into novel neural stem cells that originate new neurons [49]. An additional parallel with humans is the evidence that drosophila hematocytes, that are equivalent to macrophages in vertebrates, crucially participate in functional recovery after injury [50]. In this respect, the CNS functional restoration in the adult flies involved the glial response and included the JNK pathway activation. Among interesting mechanisms, a pivotal role in dendrite regrowth of drosophila after injuries was played by miR-87, downregulating the transcriptional repressor Tramtrack69 [51]. Furthermore, the signaling activated by the mechanosensitive Piezo non-selective cationic channels, permeable to Ca^2+^, inhibited axon regeneration in drosophila sensory neurons [52], highlighting another evolutionarily conserved mechanism.

Drosophila has been demonstrated to display neuroregenerative ability after penetrating traumatic brain injury, which is more significant in young when compared with aged flies [53]. In this model [54], the new neurons and glia appeared functional and well connected; indeed, there is a recovery of locomotion within 14 days after injury. Remarkably, the neurogenesis in the central brain differs from that in the optic lobe, where there is no proliferation of glia [55]. In addition, cells with cytoplasmic transcription factor deadpan (dpn) were identified in the optic lobe where the translocation into the nucleus of dpn following injury was coupled with neurogenesis. Since the neural progenitor gene asense (ase) was not upregulated, type I neuroblasts expressing dpn in the cytoplasm has been proposed as quiescent neuronal progenitors in the optic lobe [55]. On the contrary, dpn is missing in the healthy and uninjured central brain of flies, while ase was upregulated by injury [53]. The ase-expressing cells were found in close proximity to dpn-expressing cells, indicating that the central brain and optic lobes may undertake, at least in part, a different regenerative plan [56].

Undeniably, regeneration is a complex process that depends on injured/damaged tissue/organ, consisting of different stages. Photocontrol of specific engineering neurons by optogenetics enables the development of promising clinical neuroregenerative strategies for replacing degenerated functions or delivering pro-survival signals, since optogenetics can mix spatial and temporal light stimulation with genetic engineering to stimulate cells or tissues during a specific development phase of degenerative disease [57]. In this respect, genetically modified damaged neurons of drosophila have been shown to undertake their regeneration pathways and regulate their growth direction after stimulation with blue light [58]. In particular, the optogenetic activation of both the Raf/MEK/ERK (optoRaf) and AKT (optoAKT) signaling enhanced axon regeneration in injured neurons, but only optoRaf improved dendritic branching in CNS and the peripheral nervous system. Accordingly, it was recently reported that the optogenetics approach may induce beneficial trophic effects in a fly genetic model for parkinsonism [59]. Therefore, because of its simple genetic manipulation, *D. melanogaster* represents an ideal animal model to expand research in optogenetics and provide proof-of-concept studies.

## 3. Conclusions

Expanding new strategies in the pathophysiology of CNS is a daily challenge. Certainly, the translation of eye research to CNS and deciphering the role of immune cells in these two systems could improve our understanding and, potentially, the treatment of CNS diseases. Evaluating the impairment of the visual system at early stages to provide biomarkers of neurodegeneration is gaining attention, since it could help to test the efficacy of neuroprotective treatments and identify possible therapeutic strategies. In this context, manipulating signaling pathways that lead to neuronal regeneration offers an excellent opportunity to substitute damaged cells and, thus, restore the CNS functionality. The regenerative ability of vertebrate models, such as zebrafish, is particularly appealing. In addition, the fruit fly is an ideal and alternative animal model for regenerative studies due to its high degree of conservation with vertebrates and the broad spectrum of genetic variants achievable. Furthermore, a large part of the drosophila brain is dedicated to sight, thus offering the possibility of studying common mechanisms of the visual system and the brain at once. On the other side, zebrafish and drosophila are evolutionarily distant from mammals, and far from human complexity, representing the most significant limitation in their use. CNS anatomy differences, the less complex immune system, and the possibility that they could have a different response to stimulating-regeneration drugs imply that results should be verified on more biologically complex organisms. Indeed, the neuroregeneration pathways that work in other species, but not in mammals, may also represent a key confounding factor. In this respect, the validation of proof-of-concept results for future therapeutics would need, for instance, the comparison of treatment responses between fish, flies, and humans and/or further studies about translational biomarkers that bridge these different species. However, undeniably, the knowledge acquired from these alternative models may offer several starting points to manipulate specific well-conserved signal pathways of interest in human regeneration after injuries or during pathologies.
